# Relationship between Metabolic Syndrome and History of Cervical Cancer among a US National Population

**DOI:** 10.1155/2013/840964

**Published:** 2013-01-21

**Authors:** Eribeth K. Penaranda, Navkiran Shokar, Melchor Ortiz

**Affiliations:** ^1^Department of Family and Community Medicine, Paul L. Foster School of Medicine, Texas Tech University Health Sciences Center, 9849 Kenworthy Street, El Paso. TX 79924, USA; ^2^Department of Biostatistics, Paul L. Foster School of Medicine, Texas Tech University Health Sciences Center, 9849 Kenworthy Street, El Paso. TX 79924, USA

## Abstract

The metabolic changes present in the metabolic syndrome (MetS) have been associated with increased risk of pancreatic and colon cancers; however, there is little information about the association between MetS and cervical cancer risk. We performed a case-control study using data from the National Health and Nutrition Examination Survey (NHANES) between 1999–2010. We identified women 21 years of age and older, of which an estimated 585,924 (2.3% of the sample) self-reported a history of cervical cancer (cases). About half (48.6%) of cases and 33.2% of controls met criteria for MetS. Logistic regression analysis showed increased odds of history of cervical cancer among women with MetS (OR = 1.9; 95% CI 1.06, 3.42; *P* value ≤ 0.05) for the risk of history of cervical cancer among women with MetS while adjusting for other known risk factors (high number of lifetime sexual partners, multiparty, history of hormonal contraceptive use, and history of smoking) (AOR = 1.82; 95% CI 1.02, 3.26; *P* value ≤ 0.05). In this US surveyed population we found increased odds of history of cervical cancer among subjects with MetS.

## 1. Introduction

Cervical cancer is the second most common cancer in women worldwide, killing approximately 274,000 women in 2008, most of them in developing countries [1]. In the USA, cervical cancer cases are declining [2], but every year there continue to be about 12,000 incident cases and 4,000 deaths from this disease [3].

The causal relationship between infection with the human Papilloma Virus (HPV) and the development of cervical cancer has been established in the last two decades [4]. Out of the approximately 150 identified genotypes of HPV, 12 have been classified as category 1 carcinogens, meaning that there is sufficient evidence to determine that they cause cancer in humans [5]. Some of those types are linked to cancers other than cervical, namely, vulvar, vaginal, perianal, penis, and oropharyngeal [5]. Serotypes 16 and 18 are responsible for about 70% of all the cervical cancer cases [6, 7], but HPV 16 alone causes at least half of all cervical cancers [8]. HPV 16 is also less associated with adenocarcinoma than with squamous cell carcinoma of the cervix, which makes up more than 95% of all cervical cancers [9, 10]. HPV infection is highly prevalent among sexually active young women [6, 11], but the large majority (more than 90%) of HPV infections will clear over a few years [12]. The transition from infection with one of the oncogenic types of HPV to the immediate precursor of cervical cancer (cervical intraepithelial neoplasia 3 (CIN 3)), to invasive cancer may take 20–30 years [13, 14]. Although there are some established behavioral factors that increase the risk of developing cervical cancer in the presence of persistent infection with the HPV, such as tobacco use [15], history of high number of sexual partners [16], multiparity [16], and use of hormonal contraceptives [17], host and virulence factors that lead to persistent infection versus clearance of the HPV remain largely unknown. 

The metabolic syndrome (MetS) is a cluster of risk factors for cardiovascular disease and type 2 diabetes mellitus having hyperinsulinemia as the underlying feature [18]. The harmonized criteria define it as having three or more of the following components: central obesity, hyperglycemia, hypertriglyceridemia, hypertension and low HDL [18]. There is mounting epidemiological data that suggest that the presence of MetS increases the risk of certain cancers such as prostate, colon, and pancreas [19–23]. Theories to explain this increased risk propose that the chronic inflammation and oxidative stress associated with some of the components of MetS as well as synergism among the metabolic disarrangements that make up MetS increase carcinogenesis more than each individual component alone [21]. There is little information about the association between MetS and cervical cancer risk and there have not been studies assessing their relationship while adjusting for established risks factors for cervical cancer. 

## 2. Materials and Methods

### 2.1. Study Design and Population

We conducted a case-control study using data from the population-based National Health and Nutrition Examination Survey (NHANES) from 1999 to 2010 [24]. This survey uses a stratified, multistage, cluster sampling design to obtain a representative sample of the noninstitutionalized civilian US population and collects questionnaires, physical measurements, and body fluids samples. All participants provided written informed consent and NHANES was approved by the National Center for Health Statistics Institutional Review Board [24]. Between 1999 and 2010, 16,617 women over the age of 21 years completed the NHANES interview and examination. The current analysis excluded participants with missing data for MetS risk factors (*n* = 9, 738) and main covariates: lifetime partners ≥10, parity ≥2, history of hormonal contraceptive use, and history of smoking ≥100 cigarettes in a lifetime (*n* = 3, 365). The remaining 3,514 participants represented a population of 26,393,229 women.

### 2.2. Demographics and Covariates

Demographic information on age, sex, ethnicity, education (Diploma/GED or greater), marital status (currently married), alcohol status (three or more drinks per day), and health status (Good rated health (good, very good, or excellent)) was self-reported. Pregnancy at exam was defined as a positive lab pregnancy test or self-reported pregnancy at exam. High number of lifetime sexual partners (≥10) was defined as a response of 10 or more when asked the following questions: “In your lifetime… with how many men have you had sex” (NHANES 99-04), “… with how many men have you had vaginal, anal, or oral sex” (NHANES 05-08), or “… with how many men have you had vaginal sex” (NHANES 09-10). Multiparity was defined as a response of two or more for the following questions: “number of pregnancies resulting in live births,” (NHANES 99-04), or “number of vaginal deliveries” (NHANES 05-10). History of birth control use was defined as “yes” to “Ever taken birth control pills.” History of smoking was defined as the response of “yes” to “Smoked at least 100 cigarettes in life.”

### 2.3. Definition of Cervical Cancer and MetS

Cervical cancer cases were defined as self-reported positive history of cervical cancer. Controls were subjects without a history of cervical cancer. MetS was defined according to the harmonized criteria for MetS [18] as three or more of the following components: increased waist circumference, hypertension, hypertriglyceridemia, hyperglycemia, and low HDL. Increased waist circumference was defined as a waist circumference measure of ≥88 cm, which is the cut-off for women in the U.S. [18]. Hypertension was defined as self-report of having the diagnosis of hypertension or being on blood pressure medications or one systolic blood pressure reading of ≥130 mm Hg or one diastolic blood pressure reading of ≥85 mm Hg. Hypertriglyceridemia was defined as triglyceride serum levels of ≥150 mg/dL or use of fenofibrate or gemfibrozil medications. Hyperglycemia was defined as self-report of having the diagnosis of diabetes mellitus or being on medications for diabetes mellitus or the measurement of fasting glycemia serum levels of ≥100 mg/dL or glycohemoglobin of ≥6.5%. Low HDL was defined as serum levels of ≤50 mg/dL [25].

### 2.4. Statistical Analyses

All data management and analyses described were performed using SAS 9.2 software (Statistical Analysis System; SAS Institute; Cary, NC, USA) and were calculated while accounting for NHANES weights, strata, and cluster design. To test for the categorical association between MetS, demographics, and covariates with cervical cancer status, Wald chi-square was performed using SAS survey freq procedure. Logistic regression analysis was also performed using the SAS survey logistic procedure to determine an association between having MetS and cervical cancer, with and without adjusting for covariates.

## 3. Results

Table 1 shows MetS components, covariates, and demographics by cervical cancer status. An estimated 585,924 (2.3%) of women in this sample reported a history of cervical cancer. Although individual components of MetS and MetS itself were more common in cases than controls, no significant differences were observed (*P* values > 0.05). An estimated 48.6% of cases had MetS, compared with 33.2% of controls (*P* = 0.0768). A significantly higher proportion of cases than controls had 10 or more lifetime partners (37.3% versus 23.8%; *P* = 0.0307) and had smoked (69.2% versus 46.6%; *P* = 0.0075). For demographic factors, there were no significant differences between cervical cancer cases and controls except for self-rated health status: more controls had good health rating than cases (84.5% versus 71.0%; *P* = 0.0360).

Logistic regression analysis (Table 2) revealed that the cervical cancer cases had higher odds of MetS in both unadjusted (OR = 1.91, 95% CI (1.06, 3.42) *P* = 0.0309), and adjusted for covariates (AOR = 1.82, 95% CI (1.02, 3.26) *P* = 0.0428). When modeled alone, none of the individual components of MetS was significantly associated with cervical cancer (*P*'s > 0.05). History of smoking was the only significant covariant (OR = 2.16, 95% CI (1.11, 4.21) *P* = 0.0232, for increased odds of cervical cancer among women with MetS. High number of lifetime sexual partners remained significant in the adjusted model.

## 4. Discussion

In this US population we found that women with MetS had a twofold increased odds of history of cervical cancer compared to women without MetS. This was seen before and after adjusting for other risk factors for cervical cancer, namely, high number of lifetime sexual partners, multiparity, history of hormonal contraceptive use, and history of smoking. These results suggest that MetS may play a role in virus-host interactions needed for infection for HPV to become persistent, which is a necessary factor for the development of cervical cancer. To our knowledge, ours is the first study addressing the association between MetS and risk for cervical cancer while adjusting for the most known risk factors for cervical cancer development. We did not control for the presence of HPV infection since it is known to be a sine qua nonrisk factor for cervical cancer development [26].

A recent large longitudinal prospective study by Ulmer et al. [27] found an increased risk for cervical cancer in the presence of MetS of 26%, although smaller than the 91% increased odds found in our study, these positive associations aligned with the increasing body of evidence of the relationship between metabolic disarrangements seen in MetS and carcinogenesis. Epidemiologic studies have shown evidence that clustering MetS components increased the carcinogenic effect for colorectal cancer development and mortality compared with each individual risk factor [28, 29]. Our findings align with this theory of synergism among the components of the MetS since none of the individual MetS risk factors was associated with the history of cervical cancer, but when clustering at least three components, this association became significant and remained significant when adjusting for other risk factors for cervical cancer. 

Some individual components of the MetS have been recognized as carcinogenic. The molecular and cellular mechanisms through which obesity, hyperglycemia, and hypertriglyceridemia promote cancer development are clearer than the mechanism linking HDL and hypertension to cancer. For *Hyperglycemia, *we, like Ulmer et al. [27] found no association between hyperglycemia and cervical cancer, however, other authors have reported a positive association [30–33]. Hyperinsulinemia resulting from hyperglycemia promotes carcinogenesis indirectly through increasing circulating free insulin-like growth factor-1 (IGF-1) [34]. The risk of cancer has been found to be higher among people with raised concentrations of IGF-1 [34, 35]. Furthermore, receptors for insulin and IGF-1 are expressed in most cancer cells. The insulin receptor activating signaling pathways is capable of stimulating cancer cell proliferation, protection from apoptotic stimuli, invasion and metastasis; it also stimulates normal cells like vascular smooth muscle cell proliferation and migration needed for cancer growth [36–38]. Impaired glucose regulation and hyperglycemia also accelerate cancer cell growth. This has been demonstrated by an increase in the cancer cells glucose transporting proteins (e.g., GLUT-1) to respond to their high glucose demands [39]. *Hypertriglyceridemia* has been associated with higher occurrence of cervical cancer [30, 40] as well as colon, respiratory tract, kidney, melanoma, and thyroid cancers [41]. In our study, we did not find such association. The best understood mechanism through which elevated triglycerides promote cancer cell proliferation and their anti-apoptotic activity is via the generation of reactive oxygen species and oxidative stress that cause DNA damage [21]. Regarding *Low HDL*, to our knowledge, ours is the first study reporting on the association of cervical cancer with low HDL level; a previous study [42] found a null association with total cholesterol. 

*Obesity,* either defined as increased waist circumference or increased body mass index, has been linked to an increased incidence of several cancers as well as to a higher mortality secondary to those cancers [43, 44]. Like Green et al. and Ulmer et al. [26, 27], we did not observe any associations between cervical cancer and being overweight. Tulinius found a negative association [30], whereas other authors have found a positive association [45–48]. Besides energy storing function, the adipose tissue is an endocrine organ that regulates the function of hormones such as adipokines, leptine, plasminogen activator inhibitor-1, tumor necrosis factor alpha, and androgenous sex steroids among others. Dysfunction in the regulation of these hormones and lack of ability of fatty cells to store the extra free fatty acids seen in obesity leads to chronic inflammation [49] and carcinogenesis [50]. In accordance with this notion, an increase of adipokines and inflammatory markers has been found in older women with persistent HPV infection [51, 52]. We, like Tulinius et al. [30], found no association between cervical cancer and *hypertension*; however, Ursin has described a positive association [31]. 

History of smoking more than 100 cigarettes in a lifetime [53] was the cervical cancer risk factor with higher impact in the odds for cervical cancer in the presence of the MetS: OR = 2.16, 95%CI (1.11, 4.21). A meta-analysis found that smoking increased risk for squamous cell: OR = 1.95, 95% CI (1.43–2.65), but not adenocarcinoma of the cervix; this risk was inversely related to the age of starting smoking and directly related with the number of cigarettes smoked per day [15]. The fact that we found an even higher increased risk in the presence of MetS may suggest that MetS accounts for an additional increased risk.

The main limitation of our study is that we do not have information about the timing of the occurrence of metabolic syndrome in relation to the diagnosis of cervical cancer. Therefore, the associations found in our analysis may have been overestimated if the MetS components were not present at the time of diagnosis of cervical cancer. Nevertheless, we believe that both cervical cancer and insulin resistance/obesity may have coexisted in most of our subjects since both are insidious conditions that peak in the fourth and fifth decade of life [54, 55] and the mean age of cervical cancer diagnosis in our population was 42.8 years. Another limitation is the diagnosis of cervical cancer was determined by self-report; subjects may have had underreported or overreported their history of cervical cancer due to recall bias or because they report other cervical cancer abnormalities (e.g., ASCUS) that are not cancer, as a positive history. Although the accuracy of self-reporting screening for cervical cancer has been low [56], there is no data on the accuracy of reporting cervical cancer in the USA. Another limitation is that the surveys do not give information about the histological type of the cervical cancer (squamous cell carcinoma versus adenocarcinoma), thus limiting the ability to determine associations between each of the cervical cancer types and the MetS, however, we assume that this population follows the worldwide histologic distribution for cervical cancer in which more than 95% are squamous cell type [10].

## 5. Conclusion

We found increased odds of MetS among women with history of cervical in this US national population but no association between the individual components of the MetS and cervical cancer, suggesting that synergism among the MetS components may play a role in carcinogenesis. Thus, addressing the MetS should be part of the strategies to combat cervical cancer in the USA. Further studies are needed to corroborate these findings, ideally in a large longitudinal study that allows the collection of data of other cervical cancer risk factors and cervical cancer histologic types. More work is also needed, possibly epidemiological studies aimed to evaluate the risk of developing other cancers among individuals with the MetS to assess the additive or synergistic behavior of the individual components of the MetS in carcinogenesis. 

## Figures and Tables

**Table 1:** Characteristics of study participants by history of cervical cancer.

Characteristics	Controls (*n* = 25 , 807, 304)	Cases (*n* = 585, 924)	Wald chi-square *P* value
Age (mean ± SD, yrs)	42.4 (0.24)	42.8 (0.45)	0.7449
Years since cervical cancer diagnosis (mean ± SD)	—	14.6 (0.51)	
Age ≥ 40 years (%)	60.3	55.7	0.4938
Race			
Non-Hispanic White (%)	69.3	82.7	0.0868
Mexican American/Other Hispanic (%)	13.5	7.3	
Non-Hispanic Black (%)	12.2	6.2	
Other races and multiraces (%)	4.9	3.8	
Diploma/GED or greater (%)	82.8	78.1	0.3663
Married (%)	65.2	52.3	0.0590
Increased waist circumference (%)	60.4	65.7	0.5094
Hypertension (%)	39.8	45.0	0.4376
Hypertriglyceridemia (%)	25.3	35.3	0.1914
Hyperglycemia (%)	29.2	34.5	0.3906
Low HDL (%)	36.4	41.0	0.5128
MetS (≥3 components) (%)	33.2	48.6	0.0768
Alcohol use (≥3 drinks for day) (%)	27.2	36.8	0.2247
Pregnant at exam (%)	4.9	4.7	0.9362
Good rated health (%)	84.5	71.0	0.0360
High number of lifetime sexual partners (≥10) (%)	23.8	37.3	0.0307
Multiparity (≥2 births) (%)	62.1	62.5	0.9482
History hormonal contraceptive use (%)	84.0	91.5	0.0755
History of smoking (≥100 cigarettes in a lifetime) (%)	46.6	69.2	0.0075

MetS: metabolic syndrome; GED: general education development; HDL: high density lipoproteins.

**Table 2:** Logistic regression analysis results for the presence of MetS and cervical cancer risk (*n* = 26,393,229).

	Univariate model			Multivariate model		
	OR	95% CI	*P* value	AOR	95% CI	*P* value
MetS (≥3 components)	1.91	(1.06, 3.42)	0.0309	1.82	(1.02, 3.26)	0.0428
Increased waist circumference	1.25	(0.64, 2.44)	0.5106			
Hypertension	1.23	(0.73, 2.07)	0.4296			
Hypertriglyceridemia	1.61	(0.89, 2.93)	0.1172			
Hyperglycemia	1.28	(0.75, 2.17)	0.3644			
Low HDL	1.22	(0.70, 2.12)	0.4928			
High number of lifetime sexual partners (≥10)				1.64	(0.89, 3.00)	0.1100
Multiparity (≥2 births)				1.12	(0.62, 2.02)	0.7085
History of hormonal contraceptive use				1.81	(0.68, 4.77)	0.2332
History of smoking (≥100 cigarettes in a lifetime)				2.16	(1.11, 4.21)	0.0232

MetS: metabolic syndrome. OR: odds ratios. CI: confidence interval. AOR: adjusted odds ratios.
